# Screening Suitability of Northern Hemisphere Algal Strains for Heterotrophic Cultivation and Fatty Acid Methyl Ester Production

**DOI:** 10.3390/molecules25092107

**Published:** 2020-04-30

**Authors:** Jean Claude Nzayisenga, Calle Niemi, Lorenza Ferro, Andras Gorzsas, Francesco G. Gentili, Christiane Funk, Anita Sellstedt

**Affiliations:** 1 Department of Plant Physiology, UPSC, Umeå University, S-90187 Umeå, Sweden; jean.claude.nzayisenga@umu.se; 2 Department of Forest Biomaterials and Technology, S-90183 Umeå, Sweden; calle.niemi@slu.se (C.N.); francesco.gentili@slu.se (F.G.G.); 3 Department of Chemistry, Umeå University, S-90187 Umeå, Sweden; lorenza.ferro@gmail.com (L.F.); andras.gorzsas@umu.se (A.G.); christiane.funk@umu.se (C.F.)

**Keywords:** algae, biodiesel, FAME, FTIR, heterotrophy

## Abstract

Rapid rises in atmospheric CO_2_ levels derived from fossil fuel combustion are imposing urgent needs for renewable substitutes. One environmentally friendly alternative is biodiesel produced from suitable microalgal fatty acids. Algal strains normally grow photoautotrophically, but this is problematic in Northern areas because of the light limitations for much of the year. Mixotrophic and particularly heterotrophic strains could be valuable, especially if they can be cultivated in municipal wastewater with contents of nutrients such as nitrogen and phosphorous that should be reduced before release into receiving water. Thus, the aim of this study was to screen for microalgal strains suitable for heterotrophic cultivation with a cheap carbon source (glycerol) for biodiesel production in Nordic, and other high-latitude, countries. One of the examined strains, a *Desmodesmus* sp. strain designated 2-6, accumulated biomass at similar rates in heterotrophic conditions with 40 mM glycerol as in autotrophic conditions. Furthermore, in heterotrophic conditions it produced more fatty acids, and ca. 50% more C18:1 fatty acids, as well as showing a significant decrease in C18:3 fatty acids, all of which are highly desirable features for biodiesel production.

## 1. Introduction

There are increasingly urgent needs to replace fossil fuels, which are contributing enormously to increases in atmospheric carbon dioxide, with renewable alternatives. Moreover, first-and second-generation biofuels (mainly ethanol derived from agricultural products and lignocellulose, respectively) could be replaced by third-generation biodiesel, derived from microalgae and seaweed, where microalgae are a large group of hundreds of thousands of microscopic eukaryotic species [1]. They evolved early, apparently sharing a common ancestor with plants [2], and partly for this reason have enormous diversity, including metabolic variety. Most microalgal species are autotrophic, i.e., they have photosynthetic capacity, but some species have functionally defective plastids and hence rely on the heterotrophic acquisition and catabolism of organic carbon for their survival [1,3]. Some algae can also use light autotrophically while simultaneously utilizing organic carbon sources heterotrophically, in mixotrophic modes [3]. Their great diversity suggests that many strains may have suitable characteristics for biofuel production.

At high latitudes light is scarce for six months per year, limiting for another three months, and high during the other three months. Thus, in these regions, strains with heterotrophic capacity could be of interest for biodiesel production. Microorganisms generally preferred carbon source; glucose is too expensive for viable use in this context. However, during biodiesel production large amounts of glycerol are produced, providing a highly convenient waste carbon source. In addition to carbon, large amounts of water and mineral nutrients, most abundantly nitrogen (N) and phosphorous (P), are required for microalgal cultivation. Water is scarce in many regions, but municipal wastewater (in which the most abundant nutrients are generally N and P) is generated in all inhabited areas. Thus, municipal wastewater is a sustainable growth medium for the algal production of biodiesel [4]. Moreover, production of biodiesel as a renewable source of energy and fuel from algae cultivated in wastewater does not compete with food production, since small areas of land and/or various kinds of wasteland can be used, and both wastewater and wasteland can be potentially remediated [1,5].

Biodiesel is formed from lipids through transesterification, generally with the addition of methanol, resulting in fatty acid methyl esters (FAMEs) and the by-product glycerol. Microalgae can have higher lipid contents than oleaginous plants [5], which further increases their suitability as sources of biofuel. However, their fatty acid profile is also an important consideration, as the relative amounts of saturated and unsaturated fatty acids are important determinants of the quality of the biodiesel produced [6]. Thus, the aim of this study was to assist efforts to exploit the metabolic diversity of microalgae for biodiesel production by screeningcapability of locally isolated Nordic strains [7] for heterotrophic growth and lipid production. In addition, examination of the fatty acid profiles of the most promising strains was conducted.

## 2. Results and Discussion

Sustainable and ecologically friendly technology, supported by robust research, is required for replacement of fossil fuel [8]. Our study contributes to such research by providing information on the cultivation of microalgae in wastewater using glycerol as a carbon source, for biomass production, as well as their fatty acid composition and hence suitability for biodiesel production.

### 2.1. Pre-Screening

**Figure 1:**
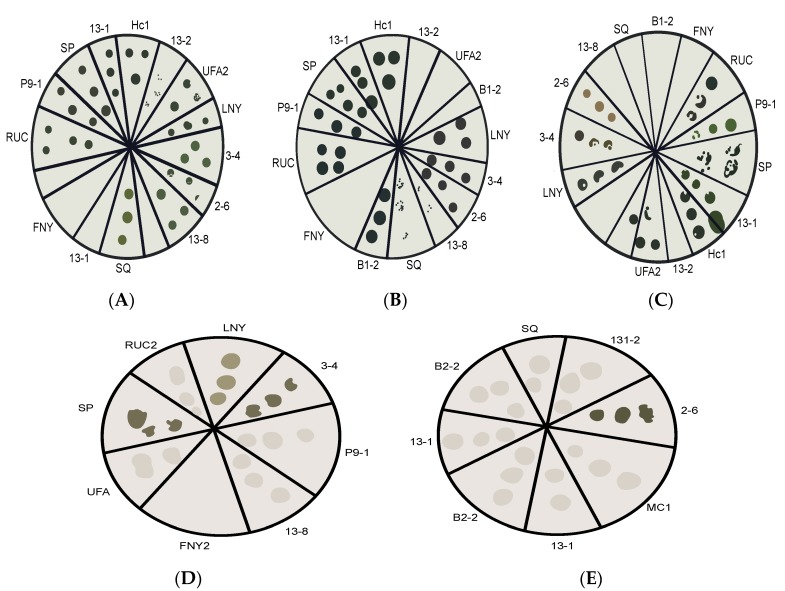
Vectorized plates from screening of auto-, mixo- and hetero-trophic growth of 14 algal strains isolated in Sweden. The strains are designated 2-6 (*Desmodesmium* sp.), 3-4 (*Coelastrella* sp.), 13-1 (*Chlorella vulgaris*), 13-2 (*Chlorella vulgaris*), 13-8 (*Scenedesmus obliquus)*, SP (*Scenedesmus obliquus*), B1-2 (*Monorapidium* sp.), UFA-2 (*Scotiollopsis reticulata*), LNY (*Chlorella vulgaris*), RUC-2 (*Desmodesmus* sp.), FNY-2 (*Ettilia pseduoalveolaris*), SQ2 (*Desmodesmus opoliensis*), MC1 (*Chlamydomonas debaryana*), P9-1 (*Micratinium* sp.). Schematic diagram showing growth of colonies of the strains on agar plates under: (**A**) autotrophic conditions, with 16 h light (100 μmL m^−2^s^−1^)/8 h dark cycles: (**B**) mixotrophic conditions with the mentioned light/dark cycles and 3 g/L glucose as a carbon source, (**C**) heterotrophic conditions with 3 g/L glucose as carbon source and (**D**) and (**E**) heterotrophic conditions with 2 g/L glycerol.

The microalgae used in this study were isolated in the Microbiorefine and Transformers consortia [7]. The pre-screening showed that 12 out of 14 algae strains were able to grow autotrophically on agar with BG11 medium (Figure 1A and Figure S1), while 11 strains grew mixotrophically with varying results (Figure 1B and Figure S1). Interestingly, nine strains showed capacity to grow heterotrophically on glucose (Figure 1C and Figure S1). However, biodiesel production must clearly be financially sustainable [9,10], and glucose is too costly for use as a carbon source. Thus, in further screening for heterotrophic capacity, the cheaper carbon source glycerol was used. Crude glycerol derived from biodiesel production has already been successfully used for algal cultivation, and in some cases increased lipid productivity by up to 370% [11]. Six tested strains were found to have the capacity to grow on glycerol, to varying degrees, confirming the high diversity of microalgae and their heterotrophic potential, which is an important characteristic for biofuel production [9]. The four strains that grew most strongly were selected for further experiments (Figure 1D,E and Figure S1). These were strains of *Desmodesmius* sp., *Coelastrella* sp., *Scenedesmus obliquus* and *Chlorella vulgaris* (designated 2-6, 3-4, SP and LNY, respectively) [9]. Furthermore, *Ettlia pseudoalveolaris* (FNY-2) was also used as an autotrophic control in further experiments.

### 2.2. Comparison of Heterotrophic and Autotrophic Algal Growth

**Figure 2:**
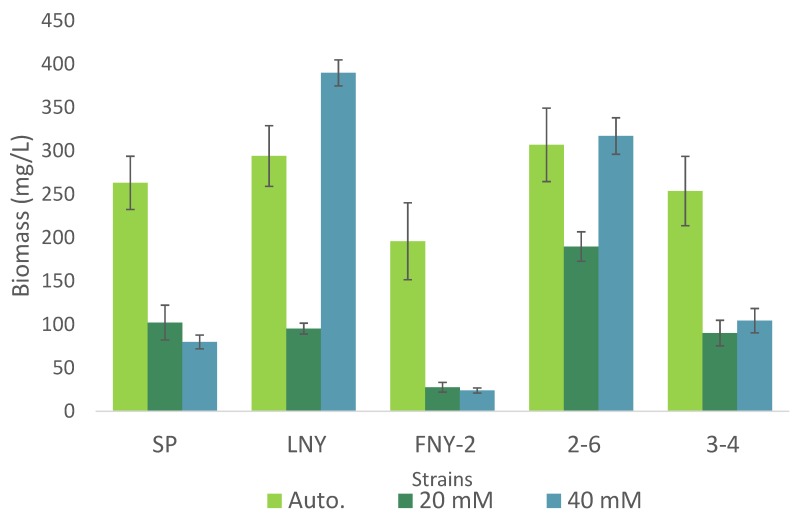
Comparison of biomass accumulation of algal strains grown under autotrophic conditions and heterotrophic conditions with 20 or 40 mM glycerol. Means ± SE, *n* = 3.

In a more detailed experiment, the five strains with the highest biomass production were grown under both autotrophic and heterotrophic conditions. The results are partly consistent with a study of autotrophic, mixotrophic and heterotrophic growth (and lipid production) by indigenous Canadian microalgae [11]. The algae designated LB1H10 in the cited study produced an equal amounts of biomass heterotrophically with 20 mM glycerol as it did autotrophically. All of the strains tested grew heterotrophically with 20 mM glycerol, but produced significantly more biomass under autotrophic conditions (Figure 2). Strains SP, FNY-2 and 3-4 also produced more biomass under autotrophic conditions than heterotrophically with 40 mM glycerol. However, strains LNY and 2-6, respectively, produced 33% and 3% more biomass heterotrophically with 40 mM glycerol than autotrophically, but the latter was not statistically significant (Figure 2) [7,11]. In the study in which these strains were initially isolated [7], their growth was found to continue up to approximately 8 days, with no pronounced stationary phase. Moreover, a *Chlorella* strain can reportedly produce more biomass under heterotrophic than autotrophic conditions, indicating that this trait may be a common physiological characteristic of algae [11].

### 2.3. Biochemical Component Analysis

**Figure 3:**
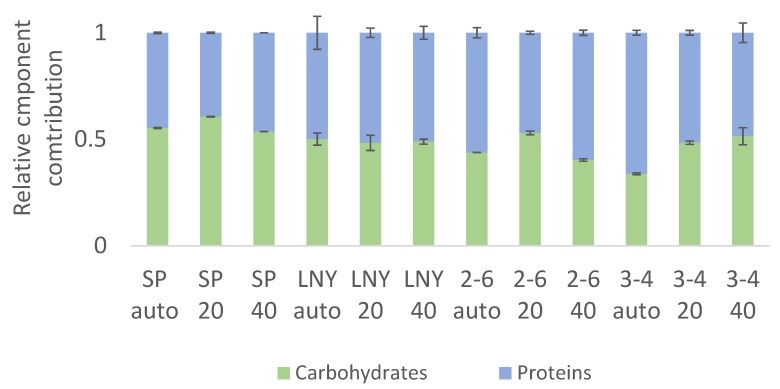
Biochemical composition (determined by FTIR) of algal strains grown under autotrophic conditions (auto), and heterotrophic conditions with 20 or 40 mM glycerol (designated 20 mM and 40 mM, respectively). Means ± SE, *n* = 3.

In FTIR spectra, peaks of specific bands can be assigned to functional groups associated with specific classes of macromolecules [12]. Accordingly, in a previous study, strong correlations between data obtained with FTIR spectroscopy and traditional analytical methods regarding both lipid contents (R^2^ = 0.99) and carbohydrate contents (R^2^ = 0.94) of microalgae [4] were found. These results provided confidence in the use of FTIR to obtain a more complete understanding of compositional differences between cells grown in autotrophic and heterotrophic conditions. In addition, a recent publication by [13] confirmed our findings. Moreover, FTIR analyses have shown, inter alia, that the carbohydrate to protein content ratio of algae changes upon illumination, confirming the suitability of FTIR analysis for such studies [14]. It has been shown by [15] that nitrogen limitation induced changes in the ratios of metabolites in freshwater algae within 7 days, and by [13] on Northern isolated algae, that FTIR spectroscopy. It has also been shown that nitrogen limitation can induce changes in ratios of metabolites in freshwater algae within 7 days [15], and that FTIR spectroscopy can be used to detect rapid changes in lipid and carbohydrate contents of algae isolated from northern regions [13]. In the algae species used in the current study, it was shown that for algae strain SP the lowest carbohydrate content was found under heterotrophic conditions with 40 mM glycerol while for strain 3-4 the lowest content was under autotrophy spectroscopy (Figure 3). In contrast, the FTIR spectroscopy results show that the carbohydrate contents of strain LNY were very similar under both heterotrophic and autotrophic conditions (Figure 3). 

### 2.4. Fatty Acid Ester Content and Composition

**Figure 4:**
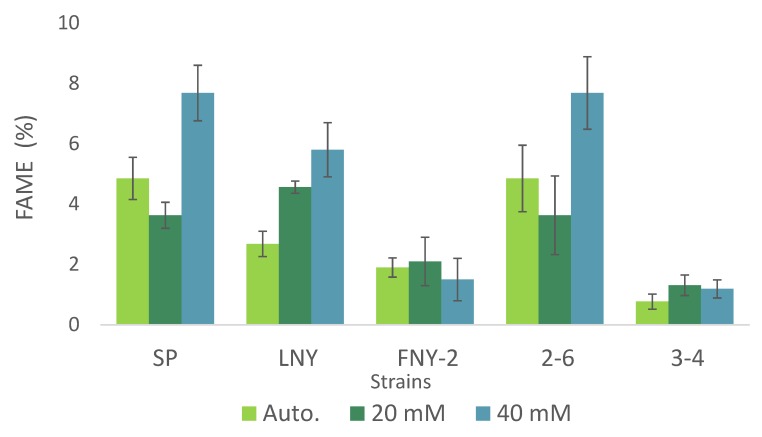
Accumulation of FAME in algal strains grown under autotrophic conditions and heterotrophic conditions with 20 or 40 mM glycerol. Means ± SE, *n* = 3.

Production of high-value molecules from microalgae has recently received increasing attention. Amongst others, lipids (especially polyunsaturated fatty acids, PUFAs) are of interest, since they can be used not only as biofuels but also in the production of cosmetics, fine chemicals and pharmaceuticals [16]. Our analysis showed that FAME accounted for approximately 5% of the biomass of strains SP and 2-6 grown autotrophically (Figure 4). Interestingly, under heterotrophic conditions with 20 mM and 40 mM glycerol, FAME accounted for 4% and 8% of their biomass, respectively, a significant difference (Figure 4). In contrast, FAME contents of strain 3-4 and FNY-2 were consistently low, ranging from 0.8 to 2% of total biomass content under all treatments, and the difference was not significantly different (Figure 4). Previous findings regarding total lipid accumulation, measured gravimetrically in autotrophically grown *Desmodesmus* strain 2-6, also indicate that its lipid production is high enough for biodiesel production [7].

Fatty acids themselves are not modified by the transesterification reaction, so the FAME composition of biofuel obtained from algae directly corresponds to their fatty acid composition. Furthermore, the FAME profile determines properties of the fuel [17] including ignition quality, heat of combustion, cold flow and oxidative stability [17]. The two most important properties of fatty acids in this respect are carbon chain length and number of double bonds.

**Figure 5:**
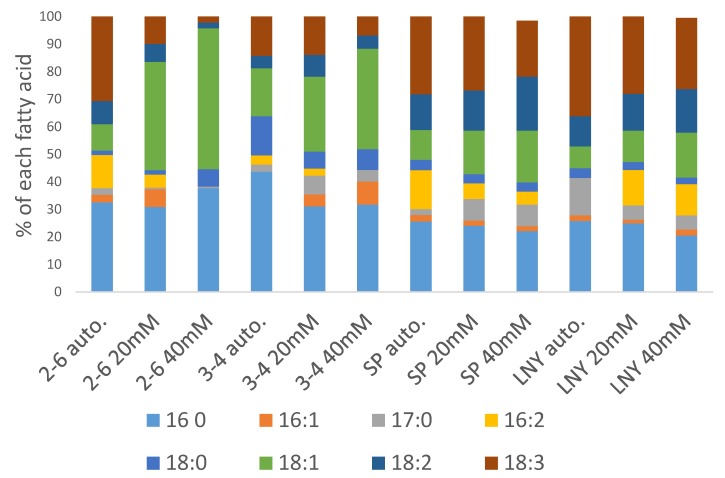
Fatty acid composition of algal strains grown under autotrophic conditions (auto) and heterotrophic conditions with 20 or 40 mM glycerol (designated 20 mM and 40 mM, respectively). Means, Standard errors are 0.1–4.2% of means, *n* = 3.

Therefore, the examination of the fatty acid profiles of our algae was conducted in more detail, and interesting results were obtained (Figure 5). It was found that strains 2-6, SP and LN-Y accumulated large proportions of 18:3 fatty acids under autotrophic conditions and heterotrophic conditions with both 20 and 40 mM glycerol. Thus, these strains seem unsuitable for biodiesel production, since 18:3 fatty acids are readily oxidized when used as a biodiesel source [17], and European standard EN 14214 specifies an upper limit of 1% for 18:3 FAMES in biodiesel [18]. However, strains 2-6 and 3-4 accumulated large proportions of desirable 18:1 fatty acids under heterotrophic conditions, and their contents were highest with 40 mM glycerol. Similarly, refs. [4,19,20] found that contents of 18:1 fatty acids were higher under heterotrophic conditions than under autotrophic conditions in *Galdiera* sp., *Chlorella* sp. and *Chlorella zofingiensis*, respectively. The use of microalgae only solely for biofuel production may not be profitable right now, but the microalgal industry could also utilize the algae as sources for high-value products [21].

### 2.5. Conclusions

The strains designated SP and 2-6 grown in 40 mM glycerol contained the highest amount of lipid content as well as fatty acid methyl esters (FAME). However, the *Desmodesmus* strain designated 2-6 grown in 40 mM glycerol contained the highest amounts of fatty acids with appropriate composition for use as biodiesel, i.e., a low levels of fatty acid 18:3 and a higher proportion of 18:1.

## 3. Materials and Methods

### 3.1. Algae Strains and Growth Conditions

In this study, 14 strains isolated following the methods within the Microbiorefine project [7], were used as experimental organisms. The strains were chosen based on earlier genus classification [7]. In Pre-screening experiments, the axenic inoculum were 2 μL of suspensions of the axenic algae grown autotrophically in 16 h light 100 μE m^−2^s^−1^ (light source Philips plant light Polylux, Leicester, UK, 8 h dark; 25 °C, up to the late exponential phase in BG11 medium [4]. The BG11 medium was only used for algae stock maintenance and for screening on agar plates. The strains were screened for heterotrophic capacity by cultivation on agar plates for 15 days containing BG11 medium and: (A) no sugars in light/dark cycles (autotrophic conditions), (B) 3 gL^−1^ glucose (Analytical reagent grade; Fischer Scientific, Leistershire, UK) or 2 gL^−1^ glycerol (99.5% purity; BDH Lab. Suppl., Poole, UK), in dark heterotrophic conditions. The optical density (OD_630_) of each inoculum was adjusted to 0.05 by dilution, according to measurements with a DU 530 spectrophotometer (Beckman, Hudson, MA, USA), as earlier described [4]. Based on results of the preliminary screening (Figure 1 and Figure S1), five strains were chosen for a more detailed study. These five algae strains were grown under the autotrophic growth condition to obtain a cell suspension for further inoculation.Triplicate sets of cells of each of the five strains were added, to an OD_630_ of 0.1, to 200 mL portions of the appropriate medium in 500 Erlenmeyer flasks, then incubated in both autotrophic and heterotrophic conditions for 8 days. The autotrophic conditions were as mentioned above, and the heterotrophic conditions were identical except that the flasks were wrapped in aluminum foil, continuously shaken (150 rpm, 25 °C), and glycerol was added at both 20 and 40 mM.

After cultivation of 8 days, cells were harvested by centrifugation using a 5417C Eppendorf centrifuge (Eppendorf, Hamburg, Germany) (4000 rpm, 6 min), and washed twice with sterile water to eliminate the original growth medium. Local municipal wastewater with the following content of total phosphorous 7.8 mgL^−1^, total nitrogen 56 mgL^−1^, BOD7 205 mgL^−1^ and COD-Cr 501 mgL^−1^ (Umeva, Umeå, Sweden). This wastewater was filtered (pore size of 10 μm, Munktell AB, Munksjö, Sweden) to separate large particles and autoclaved (121 °C, 2.5 kPa) for 20 min. A cell density of OD of 0.1 was used to inoculate a volume of 200 mL of autoclaved municipal wastewater in the 500 mL Erlenmeyer flasks. The experiments were conducted over a period of 8 days, and 10 mL portions of each culture were harvested by centrifugation for the following analysis. 

### 3.2. Assessments of Biomass

Samples of algae were washed twice with sterile water and harvested by centrifugation (3700 rpm, 6 min) as earlier described [4], stored in −20 °C until freeze-drying (−90 °C, app. 72 h), and then gravimetric determinations were performed.

### 3.3. Lipid Analysis

Lipids in triplicate samples were extracted following [22] with slight modifications [4,23]. The amounts and composition of lipids present in half of each sample were then determined using a Trace 1310 gas chromatograph (Thermo Scientific, Hägersten, Sweden), equipped with a Flame Ionization Detector (FID) and a Famewax crossbond polyethylene glycol column (30 m, 0.32 mm; Restek Corporation, Bellefonte, PA, USA), as previously described [4].

### 3.4. Trans-Methylation

Lipids were extracted and completely dried, by sparging with nitrogen gas, then transmethylated by adding 1 mL of 2% H_2_SO_4_ (p.a. quality; Merck KDAa, Darmstadt, Germany) dry methanol (p.a. quality; VWR Chemicals, Leuven, Belgium), as well as 200 μL of internal standard (Larodan AB, Solna, Sweden) (20 mg of methyl-15:0/100 mL dry methanol). Samples were then sparged for a further two minutes, then the tubes were immediately closed to avoid oxygen entering and heated for 1 h at 90 °C. Transmethylated fatty acids were extracted, and the analysis was otherwise performed as previously described [4].

### 3.5. Analysis of Proportions of Lipids, Carbohydrates and Protein Proportions

The FTIR (Fourier-transform Infrared) spectroscopy analyses were performed using a Bruker IFS 66 FTIR spectrometer equipped with OPUS 6.5 software (Bruker Optik GmbH, Ettlingen, Germany) as described in [4,24], resulting in a recording time of 90 s for each spectrum. The recorded spectra were exported as Matlab. mat files (Mathworks Inc., Natick, MA, USA) and processed using the free, open-source, Matlab-based graphical user interface available via the Vibrational Spectroscopy Core Facility at Umeå University (https://www.umu.se/en/research/infrastructure/visp/). Spectra were cut to the 830–1830 cm^−1^ (fingerprint) region, baseline corrected by asymmetrical least squares fitting (lambda = 100,000; *p* = 0.001), smoothed (Savitzky-Golay filter, first order polynomial, with a frame of three) and normalised (total area normalisation). The relative proportions of compounds were determined by evaluating the intensities (areas under peaks, i.e., integrals) of bands deemed diagnostic for a certain class of compounds. For carbohydrates, the 950–1190 cm^−1^ region was used (comprising of sugar ring breathing motions and -C-O-C- linkages), for proteins the 1575–1715 cm^−1^ region (amide I band) was used, and for fatty acids the 1715–1745 cm^−1^ region (-C=O of carboxylic moieties from lipids and fatty acids) was used. The acquired spectra were subjected to multivariate curve resolution-alternating least squares analysis, as described in [4,13].

### 3.6. Fatty Acid Analysis

The composition of fatty acid methyl esters (FAMEs) in samples was analyzed using a Trace 1310 gas chromatograph (Thermo Scientific) equipped with a FAME WAX column (30 m × 0.32 mm × 0.25 μm) and flame ionization detector. The acquired data were processed using Chromeleon 7.2 software. The analysis followed [4], except that the FAME standards (supplied by Larodan AB, Solna, Sweden) ranged from 14:0 (myristic acid) to 22:0 (behenic acid). Contents of specific fatty acids in the initial samples were calculated using the following equation:

$$\mathrm{FA}\;\mathrm{content}\;\left(\frac{\mathrm{mg}}{g}\right)=\frac{1}{100}\times \frac{\mathrm{IS}\;\mathrm{added}\;\left(\frac{\mathrm{mg}}{\mathrm{sample}}\right)\;\frac{A\text{rea of individual FAME}}{Area\;of\;C15:0\;FAME.Rel.Resp.Factor\;individual\;FAME}}{\text{amount of biomass used}}$$

## 4. Conclusions

The strains designated SP and 2-6 grown in 40 mM glycerol contained the highest amount of lipid content as well as fatty acid methyl esters (FAME). However, the *Desmodesmus* strain designated 2-6 grown in 40 mM glycerol contained the highest amounts of fatty acids with appropriate composition for use as biodiesel, i.e., a low levels of fatty acid 18:3 and a higher proportion of 18:1.

## Supplementary Materials

The supplementary materials are available online.
