# Supplementary material for: Screening Suitability of Northern Hemisphere Algal Strains for Heterotrophic Cultivation and Fatty Acid Methyl Ester Production
Source: Molecules. 2020 Apr 30;25(9):2107. doi: 10.3390/molecules25092107 (PMC7248713; doi:10.3390/molecules25092107)

Supplemental Figure 1.

Original plates from screening of auto-, mixo- and hetero-trophic growth of 14 algal strains isolated in Sweden.

The strains are designated 2-6 (*Desmodesmium* sp.), 3-4 (*Coelastrella* sp.), 13-1 (*Chlorella vulgaris*), 13-2 (*Chlorella vulgaris*), 13-8 (*Scenedesmus obliquus)*, SP (*Scenedesmus obliquus*), B1-2 (*Monorapidium* sp.), UFA-2 (*Scotiollopsis reticulata*), LNY (*Chlorella vulgaris*), RUC-2 (*Desmodesmus* sp.), FNY-2 (*Ettilia pseduoalveolaris*), SQ2 (*Desmodesmus opoliensis*), MC1 (*Chlamydomonas debaryana*), P9-1 (*Micratinium* sp.).
Schematic diagram showing growth of colonies of the strains on agar plates under: (A) autotrophic conditions, with 16 h light (100 μ mol $m^{-2}s^{-1}$)/8 h dark cycles: (B) mixotrophic conditions with the mentioned light/dark cycles and 3 g/L glucose as a carbon source, (C) heterotrophic conditions with 3 g/Lglucose as carbon source and (D) and (E) heterotrophic conditions with 2 g/L glycerol.

Supplemental Figure 1.

A B

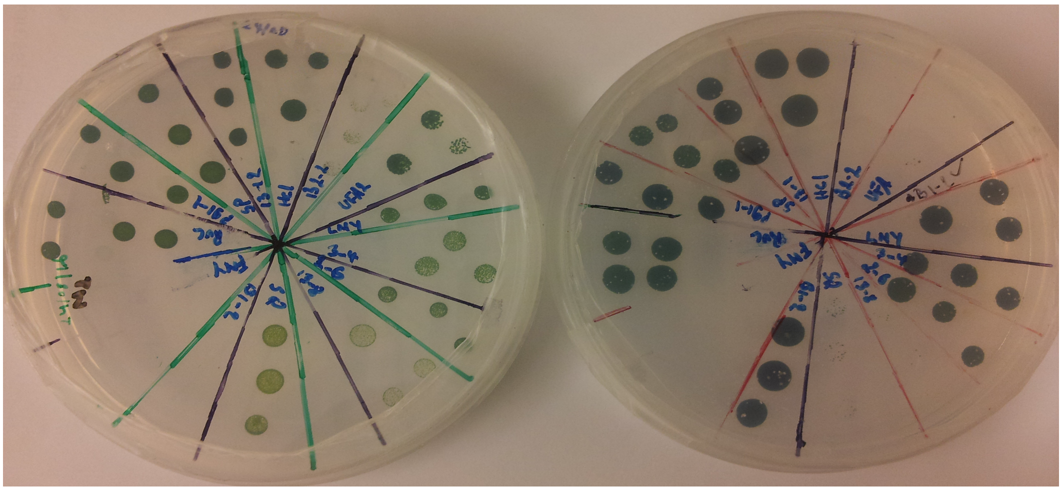

C

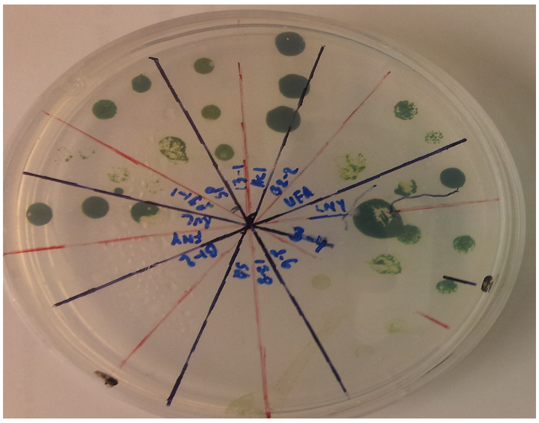

Supplemental Figure 1 cont.

D

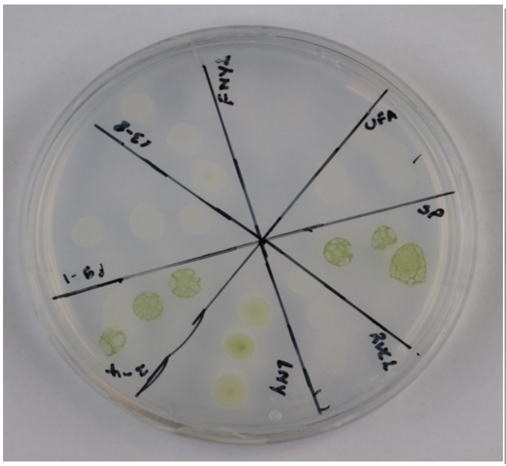

E

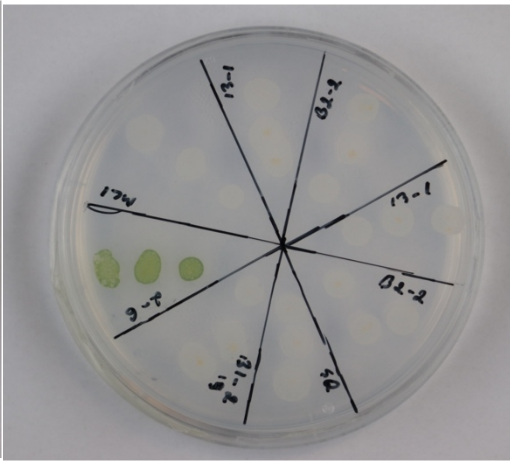

Supplement: Supplementary file 1 [file molecules-25-02107-s001.pdf]
